# 921. Clinical characteristics of hospitalized adults with herpes zoster in a Portuguese hospital: 11 years of experience

**DOI:** 10.1093/ofid/ofad500.966

**Published:** 2023-11-27

**Authors:** Eduarda Pena, Maria João M S Gonçalves, Constança Azeredo, Susana Oliveira, Sara Araújo, Luís Moura, Frederico Duarte, Sofia Jordão, Mário Guimarães, Ricardo Correia Abreu, Isabel Neves, Clara Batista

**Affiliations:** Hospital Pedro Hispano - Unidade Local Saúde Matosinhos, Matosinhos, Porto, Portugal; Hospital Pedro Hispano - Unidade Local Saúde Matosinhos, Matosinhos, Porto, Portugal; Hospital Pedro Hispano - Unidade Local Saúde Matosinhos, Matosinhos, Porto, Portugal; Hospital Pedro Hispano - Unidade Local Saúde Matosinhos, Matosinhos, Porto, Portugal; Hospital Pedro Hispano - Unidade Local Saúde Matosinhos, Matosinhos, Porto, Portugal; Hospital Pedro Hispano - Unidade Local de Saúde de Matosinhos, Matosinhos, Porto, Portugal; Hospital Pedro Hispano - Unidade Local Saúde Matosinhos, Matosinhos, Porto, Portugal; Hospital Pedro Hispano - Unidade Local Saúde Matosinhos, Matosinhos, Porto, Portugal; Hospital Pedro Hispano - Unidade Local Saúde Matosinhos, Matosinhos, Porto, Portugal; Hospital Pedro Hispano - Unidade Local Saúde Matosinhos, Matosinhos, Porto, Portugal; Hospital Pedro Hispano - Unidade Local de Saúde de Matosinhos, Matosinhos, Porto, Portugal; Hospital Pedro Hispano - Unidade Local de Saúde de Matosinhos, Matosinhos, Porto, Portugal

## Abstract

**Background:**

Herpes zoster (HZ) results from the reactivation of latent varicella-zoster virus and commonly affects older or immunocompromised patients. Clinical manifestations include painful vesicular skin eruptions that normally respect a dermatomal distribution. Prognosis is normaly favorable but a minority of patients develop complications such as generalized infection, ocular involvement, meningoencephalitis or visceral dissemination. Post-herpetic neuralgia and secondary bacterial infections are frequent complications. Preventive strategies like vaccination are important to mitigate the burden of HZ.

**Methods:**

This retrospective study reviewed the charts of all patients older than 18 years hospitalized in a Portuguese hospital with the diagnosis of herpes zoster infection diagnosis between January 1, 2012 and December 31, 2022.

**Results:**

The study included 152 patients with a mean age of 70 years old; 58% were female. In terms of clinical manifestations: 47% suffered from localized herpes zoster (thoracic dermatome in 40%) and only 11% had generalized zoster. Other manifestations included meningitis/encephalitis (7%), keratitis (n=4) and Ramsay-Hunt syndrom (n=3). About half of patients (51%) had an identified cause of imunodepression upon hospital admission: 25% had malignancy on chemotherapy and 6% had a reumatic disead on immunossupressive therapy, only 7 patients were HIV positive. Most patients (78%) were treated with aciclovir (mean duration 9 days). Post-herpetic neuralgia and secondary bacterial infections were common (35% and 20%, respectively). All-cause in-hospital mortality was 6%.

**Conclusion:**

Patients hospitalized for herpes zoster are frequently elderly people with comorbidities, especially cancer. Thoracic envolvement was most frequent in localized infection. Post-herpetic neuralgia and bacterial infections were frequent complications. Its important to implement preventive strategies such as vaccination for herpes zoster to prevent shingles and post-herpetic neuralgia in older adults, especially in those who are immunosupressed. This measure would probably help mitigate the burden of this disease in this population.

**Disclosures:**

**All Authors**: No reported disclosures

